# Ladies in the Dental Profession

**Published:** 1859-02

**Authors:** 


### LADIES IN THE DENTAL PROFESSION.

Dr. D. W. Jobson, of New York, is making efforts to induce women to enter the Dental Profession, with fair hopes of success.

We heartily second the movement. Even now we almost imagine ourself seated in what is usually termed the "chair of torture"—dreaded now no more—by our side a beautiful lady, with sweet breath and glowing cheek, her delicate arm encircling our head, our cheok resting against a bosom still more soft, looking up into eyes, so tender in their gaze—they take away all dread, and in their sympathy even divide the pain itself. With such a dentist, we would want our teeth examined every twenty-four hours.

